# Supplementary material for: Deletion of wfs1 Impairs Oligodendrocyte Precursor Cells Dorsal Distribution and Myelination Through the wfs1-hmgcs1 Axis in Zebrafish
Source: Biology (Basel). 2026 Aug 16;15(16):1402. doi: 10.3390/biology15161402 (PMC13510151; doi:10.3390/biology15161402)
Supplement: Supplementary file 1 [file biology-15-01402-s001.zip › File S1-Transcriptome sequencing data and differentially expressed genes (DEGs) in wfs1-- zebrafish at 5 dpf/2.KEGG/wfs1KOvsWT/ALL/src/dre04146.html]

dre04146


- dre:406803

- Up regulated genes

406803\_1(4.26869167669637) 406803\_2(1.03501206320206)

- Down regulated genes

406803(-2.34145157132947)

- dre:450048

- Down regulated genes

450048(-9.37553654626313)

- dre:110440136

- Down regulated genes

562105(-3.80073354274648) 110440136(-5.33161396140543)

- dre:100003383

- Up regulated genes

100003383(3.39697101268788)

- Down regulated genes

100003383\_1(-2.23974139439836)

- dre:405800

- Up regulated genes

405800(1.1459925069877)
- dre:619259

- Up regulated genes

619259(1.74407736881054)

- dre:406803

- Up regulated genes

406803\_1(4.26869167669637) 406803\_2(1.03501206320206)

- Down regulated genes

406803(-2.34145157132947)

- dre:100006589

- Up regulated genes

100006589\_1(1.74277099104316)

- dre:100093708

- Up regulated genes

100093708\_1(3.08307183149152)

- Down regulated genes

100093708(-1.95577016533434)

Close
